# Comparison of Corneal Biomechanical Properties among Axial Myopic, Nonaxial Myopic, and Nonmyopic Eyes

**DOI:** 10.1155/2020/8618615

**Published:** 2020-01-03

**Authors:** Aratchaporn Tubtimthong, Sunee Chansangpetch, Nitee Ratprasatporn, Anita Manassakorn, Visanee Tantisevi, Prin Rojanapongpun, Shan C. Lin

**Affiliations:** ^1^Glaucoma Research Unit, Faculty of Medicine, Chulalongkorn University and King Chulalongkorn Memorial Hospital, Thai Red Cross, Bangkok, Thailand; ^2^Department of Ophthalmology, University of California, San Francisco, CA, USA; ^3^Glaucoma Center of San Francisco, San Francisco, CA, USA

## Abstract

**Purpose:**

To compare corneal deformation characteristics using ultra-high-speed Scheimpflug camera (Corvis ST) in patients with nonmyopic (NM), mild-to-moderate nonaxial myopic (MM), and high axial myopic (HM) eyes.

**Methods:**

In this cross-sectional study, normal subjects aged >40 years with no history of ocular laser/surgery were classified according to axial length (AL) and spherical equivalence (SE) into three groups: (1) NM (SE > −0.50 D and AL < 26 mm), (2) MM (SE −6.00 D to −0.50 D and AL < 26 mm), and (3) HM (SE ≤ −6.00 D and AL ≥ 26 mm). Seven parameters including corneal deformation amplitude (CDA), inward/outward corneal applanation length, inward/outward corneal velocity (ICV and OCV), peak distance, and radius were measured. Pearson correlation and linear mixed-effects model were done.

**Results:**

A total of 180 eyes were recruited. 98 eyes were NM, 30 eyes were MM, and 52 eyes were HM. There were significant correlations of OCV to the degree of refractive error (*r* = 0.203, *p* < 0.001) and AL (*r* = −0.242, *p* < 0.001). After adjusting for age, sex, intraocular pressure, and corneal thickness, there was significantly higher CDA (*β* = 0.07, *p* < 0.001), faster OCV (*β* = −0.08, *p* < 0.001), and smaller radius (*β* = −0.39, *p*=0.01) in the HM group compared to the NM group.

**Conclusion:**

The higher CDA, faster OCV, and smaller radius found in the HM may suggest that these eyes have reduced ocular stiffness and may be less stable and more prone to stress.

## 1. Introduction

Myopia is one of the most common ocular problems that affect all age groups. According to recent studies and the World Health Organization (WHO) reports, refractive error is the leading cause for visual impairment and the second leading cause for visual loss worldwide [1, 2]. The prevalence of myopia is 32.9% in Southeast Asia and 16.2% in the United States [3]. The results of meta-regression analysis showed that the worldwide prevalence of myopia increased from 10.4% in 1993 to 34.2% in 2016 [3]. Approximately 20% of the myopic population has high myopia or refractive error equal to or greater than -6 diopters [4]. People with myopia, particularly those with high myopia, tend to have changes in the choroid, retina, and sclera. In severe cases, these changes may consequently lead to pathological myopia [5]. In addition, myopia increases the risk for developing cataract, glaucoma, retinal detachment and myopic retinopathy which all can lead to incurable vision loss and has become one of the major causes for visual field defects, visual impairment, and blindness [6, 7].

Axial myopia is a condition in which eyes have axial length (AL) above the norm and are too long for the whole refractive system of the eye [8]. Several structural changes in myopic eyes are associated with globe elongation. Thickness of the retina, choroid, and sclera are reduced as the axial length becomes longer [9–11]. Histologically, in axial myopic eyes, the collagenous fibers of the sclera are shown to be lengthened and disfigured from the stretching [8, 12, 13], thus affecting the globe's biomechanical properties. Since the cornea, sclera, peripapillary ring, and lamina cribrosa are formed mainly by the same extracellular matrix constituents, the corneal biomechanical properties present some insight into the action and features of collagen fibers in these other structures [14].

Corneal biomechanical parameters can be measured by two clinical devices, the Ocular Response Analyzer (ORA) and Corneal Visualization Scheimpflug Technology (Corvis ST). Corneal hysteresis (CH) is a parameter obtained from ORA and is proposed to indicate corneal viscoelasticity [15]. This parameter has been studied widely although the exact relationship between CH and viscoelastic properties of the cornea is still unknown. Corvis ST is a newly developed noncontact tonometry system device using high-speed Scheimpflug camera technology. It can record up to 4330 images per second, capturing real-time dynamic deformation of the cornea under an air puff indentation. Therefore, the device has the potential to clinically evaluate corneal deformation [16].

The aim of this study was to compare the corneal deformation characteristics of eyes with various degrees of myopia using the ultra-high-speed Scheimpflug camera. Given that myopia can be affected by other factors apart from globe size such as lens or cornea factors, our study only enrolled patients with high axial myopia with confirmed long AL.

## 2. Materials and Methods

This is a cross-sectional study that was performed at the ophthalmology outpatient clinic of the Department of Ophthalmology, King Chulalongkorn Memorial Hospital, Bangkok, Thailand. The study was approved by the Institutional Review Board of the Faculty of Medicine, Chulalongkorn University, and was conducted in accordance with the Declaration of Helsinki. Informed consent was obtained from all patients.

### 2.1. Participants

We recruited subjects who were aged over 40 years and were willing to participate in the study. Patients were excluded if any of the following conditions were present: (1) any corneal or ocular pathology (except for cataract), (2) history of corneal surgery (for example, pterygium excision or refractive surgery), (3) history of intraocular surgery, (4) pregnancy, (5) connective tissue disease, (6) inability to give consent, or (7) inability to complete the test. Subjects were divided into three groups according to the degrees of myopia and AL: (1) nonmyopia, (2) mild-to-moderate nonaxial myopia, and (3) high axial myopia. Nonmyopia (NM) was defined as having refractive error (spherical equivalence, SE) > −0.50 diopters (D) and AL < 26 mm. Mild-to-moderate nonaxial myopia (MM) was defined as having SE between −6.00 D and −0.50 D and AL < 26 mm. High axial myopia (HM) was defined as having SE ≤ −6.00 D and AL ≥ 26 mm.

### 2.2. Ocular Examinations

All subjects underwent an ophthalmic examination, including Snellen visual acuity measurement, slit-lamp examination, and Goldman applanation tonometry. Refractive error was determined by an autorefractor (Nidek AR530-A; Nidek, Gamagori, Japan). Axial length was measured by IOLMaster 500 (Carl Zeiss Meditec, Jena, Germany). Corneal biomechanical parameters were collected using Corvis ST (OCULUS Optikgeräte GmbH, Wetzlar, Germany). All ocular examinations were performed on the same day including Corvis ST imaging.

### 2.3. Image Acquisition

An ultra-high-speed Scheimpflug camera (Corvis ST) was used in this study. It can record the entire reaction of the cornea to a defined air pulse (size 3.06 mm and pressure 60 mm Hg) at the speed of 4330 images per second, obtaining a highly precise measurement of the corneal biomechanical properties. Based on the visualization of 140 images taken within 30 milliseconds during the deformation-reformation cycle after the onset of the air jet, the camera provides a detailed assessment of seven specific corneal biomechanical parameters, IOP and CCT.

**Figure 1:**
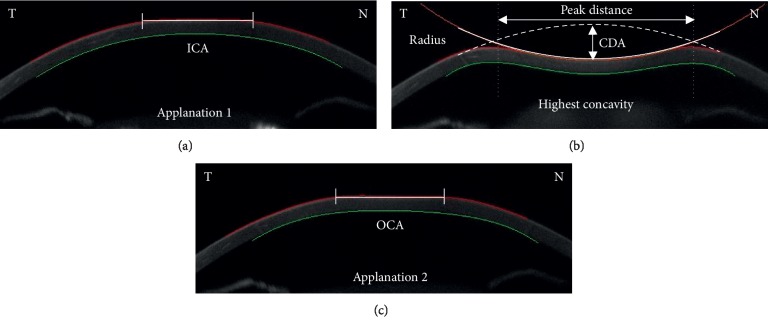
Diagram illustrating corneal deformation-reformation phases and corneal biomechanical properties measured by Corvis ST. First applanation phase (a), highest concavity phase (b), and second applanation phase (c). ICA: inward corneal applanation length; CDA: corneal deformation amplitude; OCA: outward corneal applanation length.

After an air pulse, the cornea undergoes three distinct phases: (1) first applanation, (2) highest concavity, and (3) second applanation phases (Figure 1). From the resting state, the cornea curves inward to the first flattened applanation phase (first applanation) and then continuously bends inward until reaching the maximum deformation phase (highest concavity). The cornea then recoils outward and passes the second flattened applanation phase (second applanation) to reach the full reformation, back to its resting stage.

A series of corneal parameters were analyzed automatically during a single measurement procedure as described above. The following parameters were recorded: (1) corneal deformation amplitude (CDA) (distance of the maximum corneal deformation amplitude measured from the resting state to the highest concavity at the corneal apex), (2) inward corneal applanation length (ICA) (length of the flattened cornea at the first applanation), (3) outward corneal applanation length (OCA) (length of the flattened cornea at the second applanation), (4) inward corneal velocity (ICV) (corneal velocity during the first applanation), (5) outward corneal velocity (OCV) (corneal velocity during the second applanation), (6) peak distance (PD) (distance between two bending points at the highest concavity), and (7) radius (the radius of curvature at the highest concavity) (Figure 1).

All measurements were performed by a single operator. Every data output was promptly reviewed and checked to confirm the accuracy. The quality check included the “OK” sign quality score displayed on the Corvis ST's screen, absence of image artifacts, and correct positions of the measurement lines. If the measurement did not pass the quality check, the scan was repeated again. If the quality of the image was not good by the third try, then the subject was excluded from the analysis.

### 2.4. Statistical Analysis

The data were shown as counts and percentages for categorical variables. The means and standard deviation were used for continuous variables. The chi-square test and one-way ANOVA test with the Scheffe post hoc test were used to compare clinical characteristics among the groups. Each corneal biomechanical parameter was assessed for the correlation with refractive error and axial length using the Pearson correlation. The Kruskal–Wallis test was used to compare each parameter across the groups. Then, the linear mixed-effects model was used to account for using two eyes from the same subject. The models were conducted to evaluate the effect of myopia after adjusting for age, sex, intraocular pressure, and central corneal thickness. *p* value of less than 0.05 was considered statistically significant. All analyses were done using Stata 13.0 (StataCorp, College Station, TX).

## 3. Results

A total of 180 eyes from 110 subjects were analyzed, comprised of 98 eyes from 57 subjects in the NM group, 30 eyes from 18 subjects in the MM group, and 52 eyes from 35 subjects in the HM group. There were 45 (41%) males and 65 (59%) females in the study. Significant differences were found in age, refractive error, and axial length (all *p* < 0.05) in the three groups. The mean (SD) ages were 62.2 (9.8) years in the NM group, 60.5 (8.7) years in the MM group, and 58.2 (7.9) years in the HM group. There were no significant differences in sex, laterality, best-corrected visual acuity, intraocular pressure, and central corneal thickness among the three groups. Demographic and clinical characteristics of the subjects are shown in Table 1.

**Figure 2:**
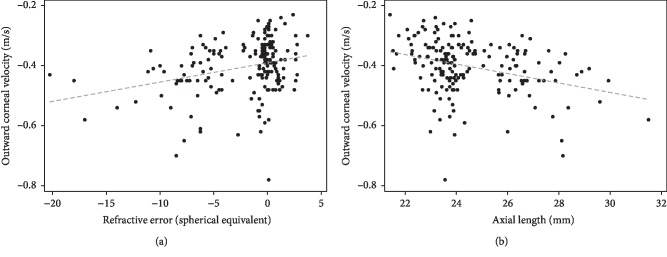
Correlation between outward corneal velocity and refractive error (a) and outward corneal velocity and axial length (b).

Among the study parameters, only OCV showed a significant positive correlation with the degree of refractive error (*r* = 0.20, *p*=0.01) and a negative correlation with the axial length (*r* = −0.24, *p*=0.001) (Figure 2).

Table 2 shows the comparison of the corneal biomechanical parameters between the 3 groups. OCV was significantly different between the three groups (*p*=0.007). Scheffe post hoc analysis revealed that the HM group was associated with significantly faster outward corneal velocity compared to the NM group (*p*=0.007). Using linear mixed-effects regression model after adjusting for potential confounding variables, the HM group demonstrated significantly greater CDA (*β* 0.07, *p* < 0.001, 95% CI 0.04 to 0.10), faster OCV (*β* −0.08, *p* < 0.001, 95% CI −0.11 to −0.05), and smaller radius (*β* −0.39, *p*=0.01, 95% CI = −0.67 to −0.11) compared to the NM group. Compared to the MM group, the HM group also demonstrated significantly greater CDA (*β* 0.05, *p*=0.004, 95% CI 0.02 to 0.09), faster OCV (*β* −0.06, *p*=0.007, 95% CI −0.10 to −0.02), and smaller radius (*β* −0.60, *p*=0.001, 95% CI = −0.96 to −0.24) (Table 3).

## 4. Discussion

Our study found a significant correlation between OCV and AL. Also, there was a significant correlation between OCV and degree of refractive error. Compared to the NM group, the HM group showed greater CDA, faster OCV, and smaller radius. The findings suggested that longer eyes may have less rigidity and limited ability to absorb external stress. Pathological changes in the scleral collagen during the progression of myopia can result in reduced scleral stiffness [17]. Corneal stroma consists of a collagenous extracellular matrix which is similar to sclera [18]. Therefore, corneal biomechanical properties, which are represented as elasticity and viscoelasticity of the cornea, may reflect the overall globe biomechanics [19].

Several changes have been documented in eyes with elongated eyeball. A prior study found a negative correlation between corneal endothelial density and axial length. It was assumed that the endothelial cells will have to flatten to cover the enlarged surface area as the axial length elongates and the anterior chamber deepens [20]. Scleral thinning has been reported in high axial myopia and pathological myopia [21–23]. The thickness of the posterior sclera of pathological myopic eyes has been reduced up to 31% compared to the sclera in nonmyopic eyes [24]. During the development of myopia, several structural changes occur such as an increase in collagen degrading enzymes and a reduction of the scleral component of collagen [18, 25]. Primary scleral contents such as glycosaminoglycan and collagen contents are reduced and disorganized. Consequently, myopic eyes become weaker and can be deformed in response to an external force [26]. The lamina cribrosa provides a support structure to the ganglion cell axons as they pass through the laminar pores within the optic disc. It is histologically composed of collagen types I, III, and IV, similar to the composition in the sclera [27, 28]. In myopia, changes in the sclera and lamina cribrosa may cause the eye to be more susceptible to IOP changes and increase the risk of glaucomatous optic nerve damage [26, 29–32].

Evidence related to the structural and biomechanical changes in the myopia has been reported. Many studies used the Ocular Response Analyzer (ORA) to assess the corneal resistance factor (CRF) and corneal hysteresis (CH). CRF is a calculated parameter that reflects the resistance of the cornea [33]. CH is a measurement of the energy absorption during the stress-strain cycle of viscoelastic composition which may represent the contribution of corneal resistance [34]. Studies from Shen et al. and Qiu et al. demonstrated that CH was significantly lower in high myopic eyes [35, 36].

The Corvis ST enables real-time visualization of the corneal responses to applied external forces. With this advantage, the device provides quantitative information regarding the magnitude and direction of corneal displacement which can reflect corneal biomechanical properties. In our study, we found that the HM group had significantly higher CDA than the NM group which suggests that the axial myopic eyes exhibited greater ocular distortion during the deformation-reformation cycle. Given that CDA demonstrates the flexibility and flaccidity of the cornea, high CDA in the HM group may indicate a reduced corneal stiffness and more instability of the ocular tissue in this condition.

We found significantly faster OCV and smaller radius in the HM group. Smaller radius indicates less central concave curvature at the highest concavity. The smaller radius in the HM group was independent to the CDA. In other words, the HM group had a small radius than the NM group even when the CDA was similar. This may imply that there is a larger bending of the area over the central cornea in the HM group. OCV represents how rapidly the cornea bends back to its resting state. We assumed that elongated myopic eyes have less rigidity to absorb external stress compared to emmetropic eyes, and thus, the globe tends to recoil backward faster. Our hypothesis is supported by previous ORA studies that showed lower corneal hysteresis in high myopia and suggested that myopic eyes had more flexibility and relative flaccidity [36–39]. Together with our data, the results suggest that the cornea in high myopia is less stiff than that in nonmyopia.

A few studies have reported on the Corvis ST parameters in myopic eyes. Most of them found, akin to our study, greater CDA, faster OCV, and smaller radius in myopic eyes [40–42]. However, these studies defined myopia based only on the refraction, without confirmation that high myopia cases were mostly due to axial elongation. Since other factors can affect the refractive error other than axial length, such as cornea or lens factors, we deliberately included only high axial myopic patients in this study to assess the biomechanical properties that are truly related to globe elongation.

Several studies have reported an increased risk of primary open-angle glaucoma associated with high myopia [43]. Jung et al. studied Corvis ST parameters among normal eyes and eyes with various glaucoma severities [44]. The results showed a tendency for faster OCV in severe glaucoma compared to mild glaucoma. After adjusting for age, IOP, and AL, their study revealed a significantly smaller radius and greater DA in severe glaucoma which were similar to the corneal deformation responses found in the HM group in our study. Our findings suggest that HM and glaucoma may share some common ocular biomechanical behavior and thus can support the epidemiological findings that show higher prevalence of glaucoma in patients with HM [45, 46]. Given that the sclera, cornea, and lamina cribrosa are connected and primarily composed of similar extracellular matrix, corneal biomechanical parameters can be regarded as global properties of the eye [10]. Corneal characteristics of faster OCV, greater CDA, and smaller radius in severe POAG may indicate greater deformability in response to increased intraocular pressure, causing the optic nerve head to become more vulnerable to damage [26, 29–31].

Prior studies showed that many factors can influence corneal parameters. CCT was reported to have a significant effect on OCV [42]. Other studies found a positive correlation between CDA and age [47]. IOP also was found to affect OCV and PD [42].

Our study also found significant correlation between OCV and AL. Therefore, to avoid any confounding effects, we statistically adjusted for these possible confounding factors. Our study has some limitations. First, our parameter did not account for scleral and periocular tissue stiffness which may influence the corneal deformation responses [48, 49]. Secondly, the age range of the recruited subjects was limited to only those aged over 40 years, so the results may not be applicable to people younger than 40 years. We controlled the age range to avoid the potential confounding effect of age on both the corneal biomechanics and extraocular tissue properties which potentially stiffens with age [48]. In addition, we did not repeat Corvis ST measurements because the air jet from the machine usually causes the subjects to be uncomfortable. However, we controlled our image quality by reviewing all images instantly after the examination and allowed only qualified images to be used in our analysis. Lastly, it should be noted that, currently, the software of Corvis ST has been updated with more available parameters, including the raw and calculated parameters, which may provide more information on ocular biomechanical properties.

## 5. Conclusions

In summary, axial myopic eyes showed significantly higher CDA, faster OCV, and smaller radius than nonmyopic eyes. Our results suggest that axial myopic eyes have a reduced ocular stiffness, indicating that the eyes are less stable and more prone to stress compared to emmetropic eyes.

## Figures and Tables

**Table 1:** Demographic and clinical characteristics of the study groups.

	Nonmyopia	Mild-to-moderate myopia	High myopia	*p* value
Age	62.2 (9.8)	60.5 (8.7)	58.2 (7.9)	**0.041**
Sex				0.796
Males	21 (36.8%)	8 (44.4%)	16 (45.7%)	
Females	36 (63.2%)	10 (55.6%)	19 (54.3%)	
Laterality				0.797
Right	46 (46.9%)	16 (53.3%)	24 (46.2%)	
Left	52 (53.1%)	14 (46.7%)	28 (53.9%)	
VA (decimal)	0.69 (0.30)	0.57 (0.30)	0.62 (0.33)	0.248
Refractive error (spherical equivalent)	0.52 (0.97)	−1.79 (1.45)	−7.61 (3.66)	**<0.001**
Axial length (mm)	23.42 (0.79)	23.66 (0.75)	27.06 (1.27)	**<0.001**
CCT (*μ*m)	541.5 (25.7)	536.1 (35.5)	543.8 (27.0)	0.480
IOP (mm Hg)	13.4 (3.6)	15.1 (3.1)	14.1 (3.2)	0.058

Data shown as mean (SD), *p* value obtained from one-way ANOVA or Kruskal–Wallis test. Data shown as *n* (%), *p* value obtained from the chi-square test. VA = visual acuity; CCT = central corneal thickness; IOP = intraocular pressure.

**Table 2:** Comparison of the corneal biomechanical parameters between the 3 groups.

	Nonmyopia		Mild-to-moderate myopia		High myopia		*p* value
	Mean	Std.	Mean	Std.	Mean	Std.	
CDA	1.127	0.124	1.133	0.111	1.144	0.101	0.684
ICA	1.775	0.053	1.766	0.097	1.780	0.045	0.601
OCA	1.712	0.327	1.694	0.399	1.597	0.384	0.168
ICV	0.146	0.016	0.148	0.016	0.144	0.014	0.524
OCV	−0.388	0.111	−0.412	0.101	−0.444	0.087	**0.007**
PD	3.201	1.242	3.467	1.241	3.221	1.337	0.625
Radius	6.766	0.691	7.043	0.760	6.603	1.195	0.114

Data shown as mean (SD), *p* value obtained from Kruskal–Wallis test. CDA = corneal deformation amplitude; ICA = inward corneal applanation length; OCA = outward corneal applanation length; ICV = inward corneal velocity; OCV = outward corneal velocity; PD = peak distance.

**Table 3:** Pairwise comparison of corneal biomechanical parameters.

	MM vs. NM				HM vs. NM				HM vs. MM			
	Coefficient	SE	*p* value	95% CI	Coefficient	SE	*p* value	95% CI	Coefficient	SE	*p* value	95% CI
CDA	0.017	0.017	0.307	−0.016 to 0.050	0.069	0.014	**<0.001**	0.042 to 0.097	0.052	0.018	**0.004**	0.017 to 0.087
ICA	−0.006	0.012	0.652	−0.030 to 0.019	0.012	0.011	0.248	−0.008 to 0.033	0.018	0.014	0.192	−0.009 to 0.044
OCA	−0.004	0.073	0.958	−0.148 to 0.140	−0.097	0.063	0.124	−0.219 to 0.026	−0.093	0.082	0.255	−0.252 to 0.067
ICV	0.001	0.003	0.653	−0.004 to 0.007	0.000	0.002	0.911	−0.005 to 0.004	−0.001	0.003	0.622	−0.007 to 0.004
OCV	−0.021	0.020	0.271	−0.060 to 0.017	−0.079	0.017	**<0.001**	−0.112 to −0.047	−0.058	0.021	**0.007**	−0.100 to −0.016
PD	0.385	0.276	0.163	−0.156 to 0.926	−0.034	0.228	0.880	−0.480 to 0.412	−0.419	0.292	0.151	−0.991 to 0.153
Radius	0.205	0.175	0.243	−0.138 to 0.548	−0.391	0.144	**0.007**	−0.673 to −0.108	−0.595	0.184	**0.001**	−0.955 to −0.235

Linear mixed-effects model adjusted for age, sex, intraocular pressure, and central corneal thickness. NM = nonmyopia; MM = mild-to -moderate nonaxial myopia; HM = high axial myopia; mild CDA = corneal deformation amplitude; ICA = inward corneal applanation length; OCA = outward corneal applanation length; ICV = inward corneal velocity; OCV = outward corneal velocity; PD = peak distance.

## Data Availability

The data used to support the findings of this study are available from the corresponding author upon request.
